# Supplementary material for: Sub-second dynamics of theta-gamma coupling in hippocampal CA1
Source: eLife. 2019 Jul 29;8:e44320. doi: 10.7554/eLife.44320 (PMC6684317; doi:10.7554/eLife.44320)
Supplement: Supplementary file 1. — Summary of experimental animals involved in the analysis. Note that TG state clustering was performed on all animals from both Hc-11 and Hc-3 data sets. Hc-11 data were specifically used for clustering analysis as well as TG state occurrence and transitions, spike-field analysis and place cell analysis. Hc-3 data were specifically used for CA3-CA1 and EC-CA1 LFP pairwise phase consistency analysis. [file elife-44320-supp1.docx]

**Supplementary File 1. Animal information.**

| **Animals** | **DataSet** | **Session** | **Maze** | **Target Region** | **Behaviors** | **Duration** |
| --- | --- | --- | --- | --- | --- | --- |
| Achilles | hc-11 | Achilles_10252013 | 1.6m linear maze | CA1 | Wake, Sleep | 9.7 hours |
| Achilles | hc-11 | Achilles_11012013 | Circular maze | CA1 | Wake, Sleep | 10.2 hours |
| Buddy | hc-11 | Buddy_06272013 | 1.6m linear maze | CA1 | Wake, Sleep | 5.8 hours |
| Cicero | hc-11 | Cicero_09012014 | 1.6m linear maze | CA1 | Wake, Sleep | 9.7 hours |
| Cicero | hc-11 | Cicero_09102014 | Circular maze | CA1 | Wake, Sleep | 10.1 hours |
| Cicero | hc-11 | Cicero_09172014 | 2m linear maze | CA1 | Wake, Sleep | 9.3 hours |
| Gatsby | hc-11 | Gatsby_08022013 | 1.6m linear maze | CA1 | Wake, Sleep | 8.4 hours |
| Gatsby | hc-11 | Gatsby_08282013 | Circular maze | CA1 | Wake, Sleep | 9.8 hours |
| ec013 | hc-3 | ec013.15 | 2.5m linear maze | EC5 EC4 EC3 CA1 | Wake | 0.2 hours |
| ec013 | hc-3 | ec013.18 | 2.5m linear maze | EC5 EC4 EC3 CA1 | Wake | 0.3 hours |
| ec014 | hc-3 | ec014.42 |  | EC5 EC3 EC2 CA1 | Sleep | 0.5 hours |
| ec014 | hc-3 | ec014.n329 |  | EC5 EC3 EC2 CA1 | Sleep | 6.5 hours |
| ec014 | hc-3 | ec014.36 | 2.5m linear maze | EC5 EC3 EC2 CA1 | Wake | 1.1 hours |
| ec015 | hc-3 | ec015.03 |  | EC5 EC3 EC2 CA1 | Sleep | 0.5 hours |
| ec016 | hc-3 | ec016.19 |  | EC5 EC4 EC3 CA1 | Sleep | 1.2 hours |
| ec016 | hc-3 | ec016.17 | 2.5m linear maze | EC5 EC4 EC3 CA1 | Wake | 0.8 hours |
| ec016 | hc-3 | ec016.19 | 2.5m linear maze | EC5 EC4 EC3 CA1 | Wake | 0.9 hours |
| gor | hc-3 | gor01-6-12 | 0.5m linear maz | CA1 CA3 | Wake | 0.2 hours |
| gor | hc-3 | gor01-6-13 | 0.8m linear maze | CA1 CA3 | Wake | 0.2 hours |
| gor | hc-3 | gor01-6-7 | 0.8 and 0.5m linear maze | CA1 CA3 | Wake | 1.5 hours |
| vvp | hc-3 | vvp01-4-9 |  | CA1 CA3 | Sleep | 1.4 hours |
| vvp | hc-3 | vvp01-4-18 | 0.8 and 0.5m linear maze | CA1 CA3 | Wake | 0.5 hours |
| vvp | hc-3 | vvp01-4-9 | 0.8 and 0.5m linear maze | CA1 CA3 | Wake | 1.0 hours |
